# ERRα protects against sepsis-induced acute lung injury in rats

**DOI:** 10.1186/s10020-023-00670-1

**Published:** 2023-06-20

**Authors:** Wenfang Xia, Zhou Pan, Huanming Zhang, Qingshan Zhou, Yu Liu

**Affiliations:** 1 Department of Critical Care Medicine, Renmin Hospital of Wuhan University, Wuhan, 430060 China; 2 Department of Cardiology, Renmin Hospital of Wuhan University, Wuhan, 430060 China; 3 Cardiovascular Research Institute, Wuhan University, Wuhan, 430060 China; 4 Hubei Key Laboratory of Cardiology, Wuhan, 430060 China

**Keywords:** Sepsis, Acute lung injury, ERRα, Apoptosis, Autophagy

## Abstract

**Background:**

Sepsis-induced acute lung injury (ALI) is associated with poor survival rates. The identification of potential therapeutic targets for preventing sepsis-induced ALI has clinical importance. This study aims to investigate the role of estrogen-related receptor alpha (ERRα) in sepsis-induced ALI.

**Methods:**

Lipopolysaccharide (LPS) was used to simulate sepsis-induced ALI model in rat pulmonary microvascular endothelial cells (PMVECs). The effects of ERRα overexpression and knockdown on LPS-induced endothelial permeability, apoptosis and autophagy were determined by horseradish peroxidase permeability assay, TdT-mediated dUTP Nick End Labeling (TUNEL) assay, flow cytometry, immunofluorescence staining, RT-PCR and Western Blotting. The rat model with sepsis-induced ALI was established by cecal ligation and puncture in anesthetized rats to verify the results of in vitro experiments. Animals were randomly assigned to receive intraperitoneal injection of vehicle or ERRα agonist. Lung vascular permeability, pathological injury, apoptosis and autophagy were examined.

**Results:**

Overexpression of ERRα ameliorated LPS-induced endothelial hyperpermeability, degradation of adherens junctional molecules, upregulation of bax, cleaved caspase 3 and cleaved caspase 9 levels, downregulation of anti-apoptotic protein Bcl-2 level, and promoted the formation of autophagic flux, while the knockdown of ERRα exacerbated LPS-induced apoptosis and inhibited the activation of autophagy. Administration of ERRα agonist alleviated the pathological damage of lung tissue, increased the levels of tight junction proteins and adherens junction proteins, and decreased the expression of apoptosis-related proteins. Promoting the expression of ERRα significantly enhanced the process of autophagy and reduced CLP-induced ALI. Mechanistically, ERRα is essential to regulate the balance between autophagy and apoptosis to maintain the adherens junctional integrity.

**Conclusion:**

ERRα protects against sepsis-induced ALI through ERRα-mediated apoptosis and autophagy. Activation of ERRα provides a new therapeutic opportunity to prevent sepsis-induced ALI.

## Introduction

Sepsis is a life-threatening multiple organ failure caused by the uncontrolled inflammatory response of the body in response to infections and non-infections. It is a common disease in the intensive care unit with a high case fatality rate (Dombrovskiy et al. 2007). For the past few years, although great improvement has been made in treatments such as hemodynamic support, anti-infective therapy, mechanical ventilatory support, and other life support, sepsis is still the main cause of death in ICU (Xia et al. 2019). Sepsis is one of the major causes of acute lung injury (ALI) and acute respiratory distress syndrome, which were accompanied by high mortality. The pathological changes in sepsis-induced ALI involve the damage or death of PMVECs and alveolar epithelial cells (Tanaka et al. 2012). Due to the unique anatomic and physiological characteristics, PMVECs can be injured not only by the influence of endovascular factors such as inflammatory factors, adenosine and angiotensin, but also by the stimulation of environmental factors such as LPS, smoking and hypoxia. Injured PMVECs develop inflammation, oxidative stress, and apoptosis, which affect the integrity of lung tight junctions and adherens junction, increase the permeability of pulmonary microvascular endothelium, lead to pulmonary edema and alveolar collapse, and eventually cause ALI.

It is well established that apoptosis, one of the several major types of cell death, is involved in the pathogenesis of sepsis-induce ALI (Jiang et al. 2020). Different from necrosis, which causes extensive intracellular damage, apoptosis represents highly regulated form of cell death that involves in tissue development and homeostasis, initiated by one of two pathways: the intrinsic pathway or extrinsic pathway. Stressed cells generate intracellular signals and activate the intrinsic apoptosis pathway, which depends on the proteases release of mitochondria with an intact plasma membrane (Galluzzi et al. 2007). The extrinsic pathway depends on the combination of extracellular ligands and cell-surface death receptors, and then causing the formation of the death-inducing signaling complex. Previous study has shown that CircC3P1 attenuated inflammation and apoptosis in ALI through modulating miR-21, thereby alleviating sepsis-induced ALI (Jiang et al. 2020). Therefore, we speculate that controlling the activation of abnormal apoptosis can alleviate sepsis-induced-ALI.

Autophagy is a regulated catabolic process of the cell that engulfs of unnecessary or dysfunctional components, playing a vital role in cellular homeostasis responses to metabolic stresses such as starved and non-starved stimuli (Ravikumar et al. 2010). Targeted cytoplasmic components are isolated and engulfed into intracellular double-membrane vesicles, known as autophagosomes, which, in time, fuses with applicable lysosomes to form autolysosomes. The targeted cytosol or organelles are digested by hydrolases in autolysosomes, degrading and recycling metabolic precursors which can be regenerated for biosynthesis and then promoting cell survival (Ravikumar et al. 2010; Mizushima et al. 2008). Hu et al. showed that activation of MTOR and subsequent inhibition of autophagy in the epithelium aggravates sepsis-induced ALI via inducing NF-κB pathway (Hu et al. 2016). In addition, previous study has shown that RAB26-dependent autophagy prevents against the degradation of adherens junctional in LPS-induced ALI in *vivo* and *vitro* (Dong et al. 2018).

Estrogen related receptor α (ERRα) is one of the earliest identified orphan nuclear receptors, named for its similarity to human estrogen receptor α in DNA binding domain (Giguère et al. 1988). Previous studies have reported that ERRα is a key transcriptional regulator of energy metabolism, and involves in glucose and lipid metabolism, mitochondrial biogenesis and metabolic adaptation of various cells, tissues and organs (Deblois and Giguère 2011; Villena and Kralli 2008). The synergistic effect of ERRα and peroxisome proliferator-activated receptor-c coactivator 1α (PGC-1α) regulates the expression of mitochondrial related genes (such as cytochrome c, ATP synthase β and superoxide dismutase) and the production of reactive oxygen species (ROS) through targeting SIRT3 (Zhang et al. 2016). The over-generated mitochondrial ROS not only leads to endothelial cell apoptosis, but also affects the integrity of vascular endothelial adhesion junction protein (Kluge et al. 2013). In addition to its known role in regulating the function of mitochondria, ERRα is increasingly considered to be a crucial regulator of immune and inflammatory pathways (Hong et al. 2013; Huss et al. 2015). It has been reported that ERRα promotes post-translational activation of autophagy via activation of SIRT1 and subsequently deacetylation of autophagy-related genes (Kim et al. 2018). Research on the role of ERRα in the modulation of apoptosis and autophagy in sepsis-induced ALI is still limited. Therefore, in this study, we aimed to investigate whether ERRα protects against sepsis-induced ALI via regulation of apoptosis and autophagy in rats.

## Materials and methods

### Animals and sepsis-induced ALI model

Male Sprague-Dawley rats with weight between 180 and 230 g were obtained from Hubei Provincial Laboratory Animal Public Service Center (Wuhan, China) and maintained at the Center of Experimental Animals of Renmin hospital of Wuhan University under specific pathogen-free conditions. All animal experiments in this research were approved by the Laboratory Animal Welfare & Ethics Committee of Renmin Hospital of Wuhan University (IACUC Issue No. 20,201,208).

The rats were randomly divided into the following four groups (n = 8–10/group): (i) sham group, (ii) cecal ligation and puncture (CLP) + vehicle group, (iii)CLP + ERRα agonist (5 mg/kg), (iv) CLP + ERRα agonist (10 mg/kg). The CLP-induced septic ALI model was established as described previously (Xia et al. 2020). Briefly, rats were anesthetized with an intraperitoneal injection of 25 mL/kg body weight of 2% pentobarbital sodium. About 2 cm midline abdominal incision was made to isolate and expose cecum which was then ligated below the ileocecal valve and puncture twice with a 20-gauge needle between the ligation and the tip of the cecum to pierce the cecum. Then extruded a small droplet of feces into the abdominal cavity. Sham control rats underwent the same operation but without CLP. 2-Phenyl-4 H-Pyrido[1,2-α]pyrimidin-4-ones, the new agonist improving the transcriptional functions of ERRα (Peng et al. 2011), was purchased from Matrix Scientific (CAS Number 16054-93-6, Columbia). Rats in the CLP + agonist group received 5 or 10 mg/kg 16054-93-6 50 min before CLP intraperitoneally, and rats in the CLP + vehicle group received the same volume of normal saline. At 24 h after CLP, the animals were sacrificed to collect lung tissues and immediately measured its weight (wet weight, W). Then, the lung tissue was dried at 65 °C for 48 h to weight its dry weight (D). The W/D ratio was used to estimate the degree of pulmonary edema.

### Cell culture and treatments

The normal rat PMVEC cell line was purchased from from Bei Na Biotechnology Research Institute (Beijing, China) and maintained in Dulbecco’s Modified Eagle’s Medium: F-12 (DMEM/F12, GENOM, China) containing 10% fetal bovine serum (FBS, ScienCell, USA) at 37°C in humidified atmosphere of 5% CO2. To knockdown ERRα, the recombinant lentiviral sh-ERRα (Target sequence, 5’-ACTCTGACTCCGTGCACATTGCTC-3’, 9.59 × 10^8^TU/ml, RiboBio Co., Ltd, Guangzhou, China) was used to infect PMVECs as described previously (Peng et al. 2011). Scramble sh-RNA was used as control. To overexpress ERRα, PMVECs were transfected with recombinant adenovirus-containing ERRα expression vector (Ad- ERRα, pAV[Exp]-EGFP-CMV > rERRα, 5.53 × 10^10^pfu/ml, Vector Builder, USA) or control vector. After 48 h, the transfection efficiency was verified using qRT-PCR and western blot. To induce ALI-related damage, transfected or non-transfected cells were treated with LPS for 12 h (Escherichia coli O26:B6, 10ug/ml, Sigma, USA). The mTOR inhibitor, rapamycin was purchased from MedChemExpress (shanghai, China) and applied to the cells for 12 h at a final concentration of 20nM before LPS-treated. The tandem RFP-GFP-LC3 adenovirus (MOI = 10, Hanbio Inc, China) were used to infect cells and observe the various stages of autophagy.

### RT-PCR

Total RNA was extracted from the lung tissue and PMVECs using Trizol (Takara, Japan). RT-PCR experiments were conducted according to the MIQE guidelines (dMIQE Group et al., 2020). A total of 1 µg of RNA was used as a template to synthesis cDNA using a cDNA synthesis kit. The RT-PCR analysis was performed using SYBR Greenbased reagent (Qiagen, USA) and StepOnePlus Real-Time PCR System (Applied Biosystems, USA) to determine the mRNA expression levels. The specific primers of ERRα and GAPDH are as follows: ERRα: forward, 5’-ACTGCAGAGTGTGTGGATGG-3’, reverse, 5’-ACGGAGTCAGAGTTGGCAAG-3’; GAPDH: forward, 5’-AGTGCCAGCCTCTCATA-3’, reverse, 5’-TCCCGTTGATGACCAGCTTC-3’.

### Western blotting

The total protein of lung tissues and cells was collected with RIPA buffer (Servicebio, Wuhan, China) with cocktail and phosphatase inhibitors (Thermo Fisher Scientific, Wuhan, China), followed by electrophoresed through 8–12% sodium dodecyl sulfate–polyacrylamide gel and then transferred to 0.45 μm PVDF membrane (Merck Millipore, USA). A total of 30 ug of protein was used for western blot experiments. After blocked with 5% milk, the membrane was immunoblotted with following primary antibodies(Except for labeled, all from Cell Signaling Technology, MA, USA) at 4 °C overnight: anti-ERRα (1:1000), anti-ZO-1 (1:1000; Abcam), anti-VE-cadherin (1:1000), anti-Occludin(1:1000), anti-JAM-A(1:1000; Abcam), anti-Sirt3 (1:1000), anti-Bcl-2 (1:1000), anti-Bax (1:1000), anti-Smac (1:1000), anti-Cytochrome c (1:1000), anti-caspase 3 (1:1000), anti-cleaved caspase 3 (1:500), anti-caspase 9 (1:1000), anti-cleaved caspase 9 (1:500), anti-LC3A/B(1:1000), anti-Beclin1 (1:1000), anti-p62 (1:1000), or anti-GAPDH (1:10000, Abcam, internal control). After incubated with the HRP-conjugated secondary antibodies at 25℃ for 1 h, the immunoblots were visualized using the ECL system. The gray value was analyzed using the Image J and calculate the relative protein level based on the density ratio of the target protein to GAPDH (internal control).

### Immunofluorescence staining

PMVECs were grown on coverslips and treated as indicated, fixed with 4% paraformaldehyde and then permeabilized 0.1% Triton X-100. After blocked with 5% BSA, Slides were stained with relevant primary antibodies (anti-ERRα antibody (1:100), anti-ZO-1 antibody (1:100), anti-p62 antibody (1:100)), and Alexa Fluor 488-conjugated secondary antibodies (1:2000; Cell Signaling Technology). After dewaxing, blocking and antigen retrieval, the paraffin-embedded lung sections were subjected to immunofluorescence staining with primary antibodies against LC3A/B (1:100), p62(1:100) and Alexa Fluor 488-conjugated and 594-conjugated secondary antibodies. Then sections were treated with DAPI (Beyotime) to stain the nuclei. All images were captured at a fluorescence microscope (Olympus).

### TdT-mediated dUTP nick end labeling (TUNEL) assay for apoptosis

TUNEL Apoptosis Assay Kit (Beyotime) was used to verify DNA fragmentation by labeling 3’-hydroxyl termini in the double-strand DNA breaks according to the instructions of manufacturer. Stain with DAPI for 5 min and the apoptotic nucleus produced red fluorescence.

### Flow cytometry

After digesting each group of cells with trypsin without EDTA, the cells were washed with cold PBS, and then resuspended in 1×Binding Buffer. Add FITC annexin V (5ul) and PI (10ul, BD, USA) to treated-cells (1 × 10^5^/100µl), which were gently mixed and incubated at 25 ° C in dark for 15 min, then add 400 µl 1×Binding Buffer to each test tube. The apoptosis was analyzed by flow cytometry within 1 h.

### Histology and immunohistochemical (IHC) analyses

Paraffin-embedded rats lung Sects. (3–4 μm thickness) were prepared by routine procedure. The sections were stained with hematoxylin-eosin to assess the degree of pathological injury by standard procedures. Lung injury, which was evaluated based on edema, neutrophil infiltration and alveolar collapse, was graded according to the percentage injury in the lung parenchyma region as previously described (Kiyonari et al. 2000). IHC assessment was performed according to the description of previous study (Taylor et al., 2006). The following antibodies were used for IHC analyses: anti-cleaved caspase 3 (1:100), anti-VE-cadherin (1:200). After incubation with primary antibodies at 4 °C overnight, the slides were then stained with HRP-conjugated secondary antibody. Five random fields of each slide were viewed under a light microscope (Olympus, Japan).

### Endothelial permeability and lung vascular permeability assay

The PMVECs permeability was analyzed by HRP permeability assay, as described previously (Wei et al. 2017). The cells were treated with LPS for 2 h, 6 h, and 12 h, then the HRP activity was measured Microplate Reader (Perkin Elmer, USA) at OD450. To assay the lung vascular permeability, Evans blue dye (50 mg/kg) was injected intravenously and the pulmonary artery was perfused with normal saline to remove intravascular Evans blue dye. After allowed to circulate for 5 min, the BALF was collected to determine the concentration of Evans blue dye by measuring the absorbance at 630 nm.

### Cell count in bronchoalveolar lavage fluid (BALF)

The trachea of the anesthetized rats was exposed. The lungs were lavaged thrice with 4.0 ml of saline (4 °C) inserted through an endotracheal tube and recycling the BALF after each 30s of lavage. The collected BALF was centrifuged at 4 ℃ for 10 min. After discarding the supernatant, the cells were resuspended with 0.3 ml PBS (pH 7.4), and the total cells were counted with cell counting plate.

### Statistical analysis

Data are expressed as the mean ± SD and were analyzed using GraphPad Prism 6 (GraphPad Software) with student’s t-test between two groups, one-way analysis of variance (ANOVA) followed by Fischer’s least significant different (LSD) test for groups of three or more, and p < 0.05 was considered statistically significant. All experiments were repeated three times as independent experiments.

## Results

### Overexpression of ERRα alleviates LPS-induced endothelial hyperpermeability in PMVECs

**Fig. 1:**
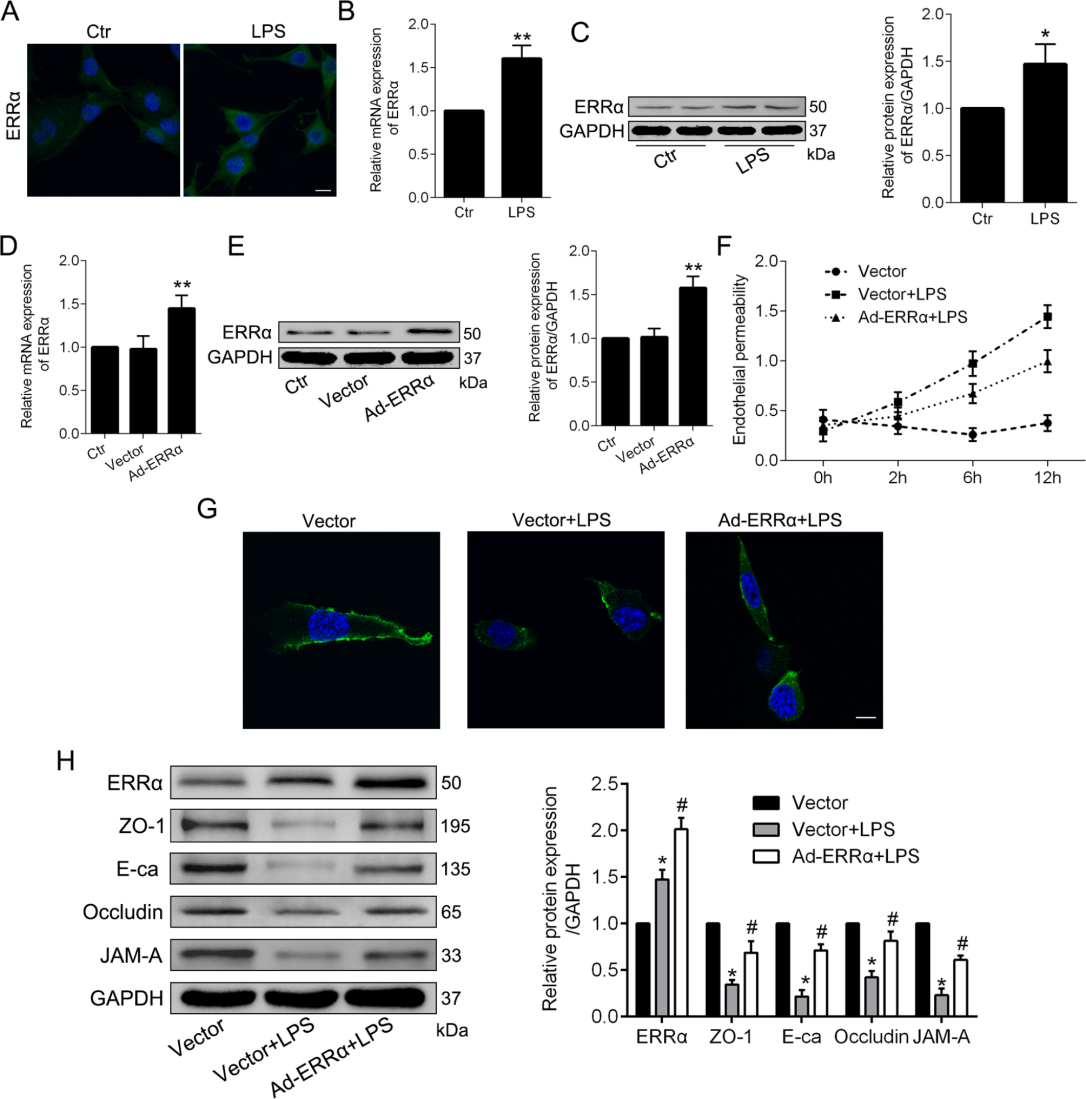
Overexpression of ERRα alleviates LPS-induced endothelial hyperpermeability in vitro. **A**. Representative immunofluorescence showing the expression of ERRα from LPS-treated PMVECs, observed by confocal microscopic images. Scale bar, 10 μm. **B**-**C**. RT-PCR (**B**), Western blot and quantitative analysis (**C**) of ERRα expression in Control and LPS groups. **D**-**E**. The levels of ERRα mRNA (**D**), ERRα protein and quantitative analysis (**E**) after transfected Ad-Vector and Ad-ERRα adenovirus. **F**. Comparison of endothelial permeability in three groups of cells after LPS treated 2, 6, 12 h. **G**. Representative confocal microscopic images showing the expression of Zo-1 in three groups. Scale bar, 10 μm. **H**. Western blot and quantitative analysis of ZO-1, Occludin, JAM-A, and VE-cadherin. Values are expressed as mean ± SD. ** P < 0.01, *P < 0.05 versus the Control or Vector group; #P < 0.05 versus the Vector + LPS group

Previous report showed that ERRα attenuated LPS-induced inflammatory response via regulating NF-κB signaling in macrophages (Yuk et al. 2015). We speculated that ERRα also has a protective effect in LPS-treated PMVECs. Treatment with LPS upregulated the levels of ERRα in cytoplasm of PMVECs (Fig. 1A), as well as the mRNA and protein expression levels of ERRα (Fig. 1B-C), which was consistent with previous studies (Xia et al.,2020; Yuk et al. 2015). To explore the regulatory role of ERRα in LPS-treated PMVECs, transfection efficiency of upregulated ERRα on PMVECs was evaluated (Fig. 1D-E). Compared with the vector cells, LPS-treated PMVECs manifested a significant increase in endothelial permeability. However, ERRα gene overexpression effectively attenuated LPS-induced PMVECs hyperpermeability (Fig. 1F). ZO-1 immunostaining revealed that overexpression of ERRα diminished the formation of intercellular gaps and degradation of adherens junctions after LPS stimulation (Fig. 1G). Western blot results showed a significant reduction in expression of tight junction proteins ZO-1, Occludin, JAM-A, and adherens junction protein VE-cadherin from LPS-induced PMVECs, which was ameliorated by promoting the expression of ERRα (Fig. 1H).

### ERRα has a protective effect in LPS-induced apoptosis in PMVECs

**Fig. 2:**
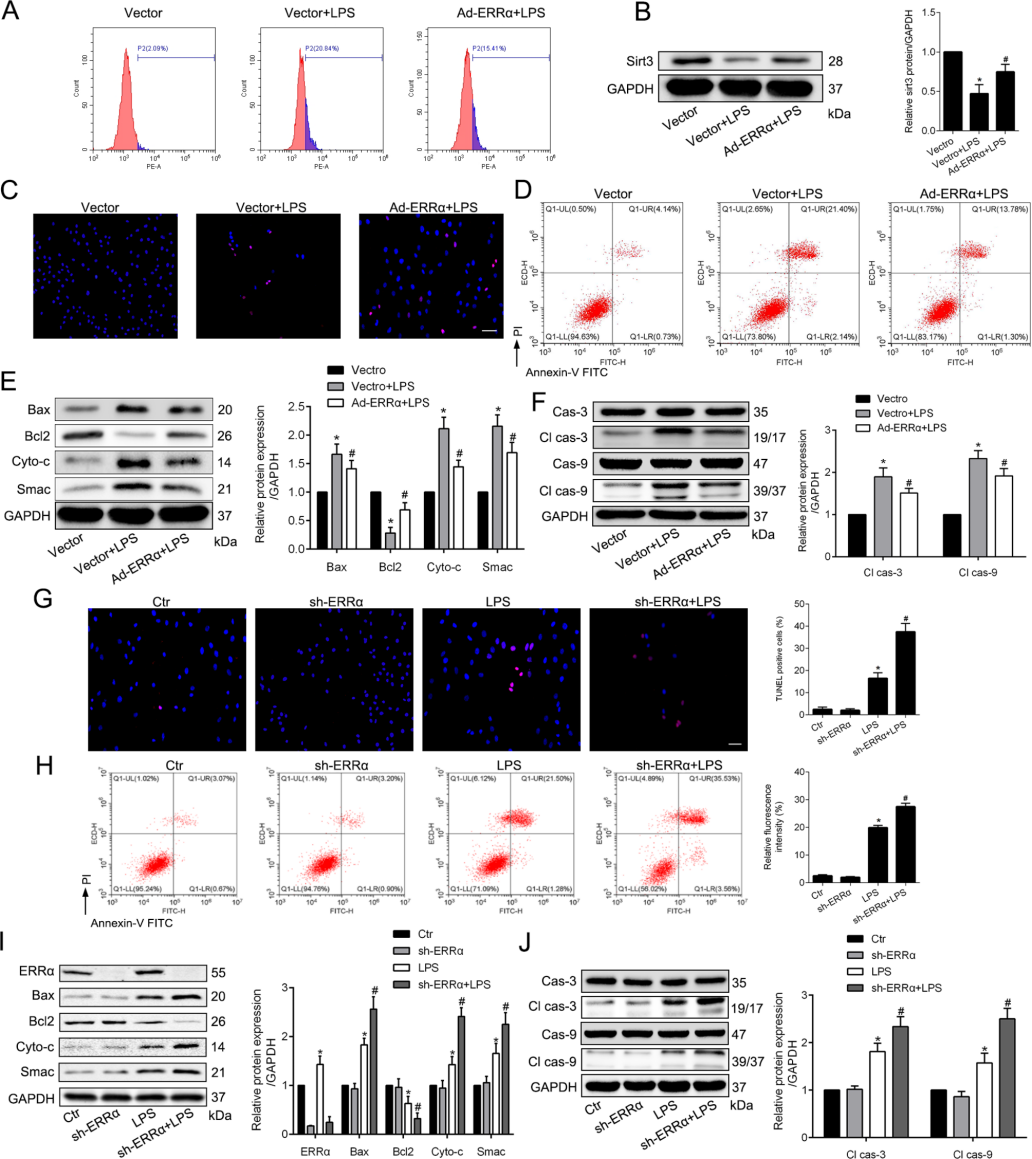
ERRα has a protective effect in LPS-induced apoptosis in PMVECs. **A**. The production of ROS in PMVECs using flow cytometry, and the shift to right of the peak represents an increase in ROS production. **B**. Representative blots and quantitative results of Sirt3 in three groups. **C**, **G**. TUNEL analysis of cells apoptosis in LPS-treated PMVECs transfected with Ad-ERRα adenovirus (**C**) or sh-ERRα lentivirus (**G**). the red nucleus is the positive. Scale bar, 50 μm. **D**, **H**. The apoptosis of cells stained with Annexin V and PI was determined by flow cytometry in LPS-treated PMVECs transfected with Ad-ERRα adenovirus (**D**) or sh-ERRα lentivirus (**H**). **E**-**F**, **I**-**J**. Representative blots and quantitative analysis showing the relative protein levels of apoptosis-associated proteins after transfected with Ad-ERRα adenovirus (**E**-**F**) or sh-ERRα lentivirus (**I**-**J**). *P < 0.05 versus the Control or Vector group; #P < 0.05 versus the Vector + LPS or LPS group

It is reported that ERRα activation could inhibit cells apoptosis through the Bcl-2/Caspase3 pathways (Huang et al. 2020) and regulate the expression of reactive oxygen species (ROS) through targeting SIRT3 (Zhang et al. 2016). To assess whether ERRα provide a preventative benefit in LPS-induced ALI, we pre-treated PMVECs with overexpression or knockdown vector of ERRα before exposed to LPS. Stimulation with LPS resulted in the production of ROS (Fig. 2A), genesis of apoptosis (Fig. 2C-D) and the decrease of Sirt3 (Fig. 2B) in PMVECs. However, the Ad-ERRα-transfected cells exhibited decreased production of ROS and apoptosis, and retained Sirt3 protein when compared with the vector transfected cells after LPS treatment. Meanwhile, we also observed that exposing PMVECs to LPS led to the degradation of anti-apoptotic protein Bcl-2 and the increased expression of apoptotic proteins, such as Bax, Smac, Cytochrome c, cleaved caspase 3 and cleaved caspase 9 (Fig. 2E-F). As expected, knockdown of ERRα further aggravated the LPS-induced PMVECs apoptosis (Fig. 2G-I). These findings indicate that ERRα overexpression significantly inhibits oxidative stress and apoptosis in the LPS-stimulated PMVECs and the deficiency of ERRα exhibits a detrimental effect.

### ERRα is essential for autophagy activation in PMVECs in response to LPS

**Fig. 3:**
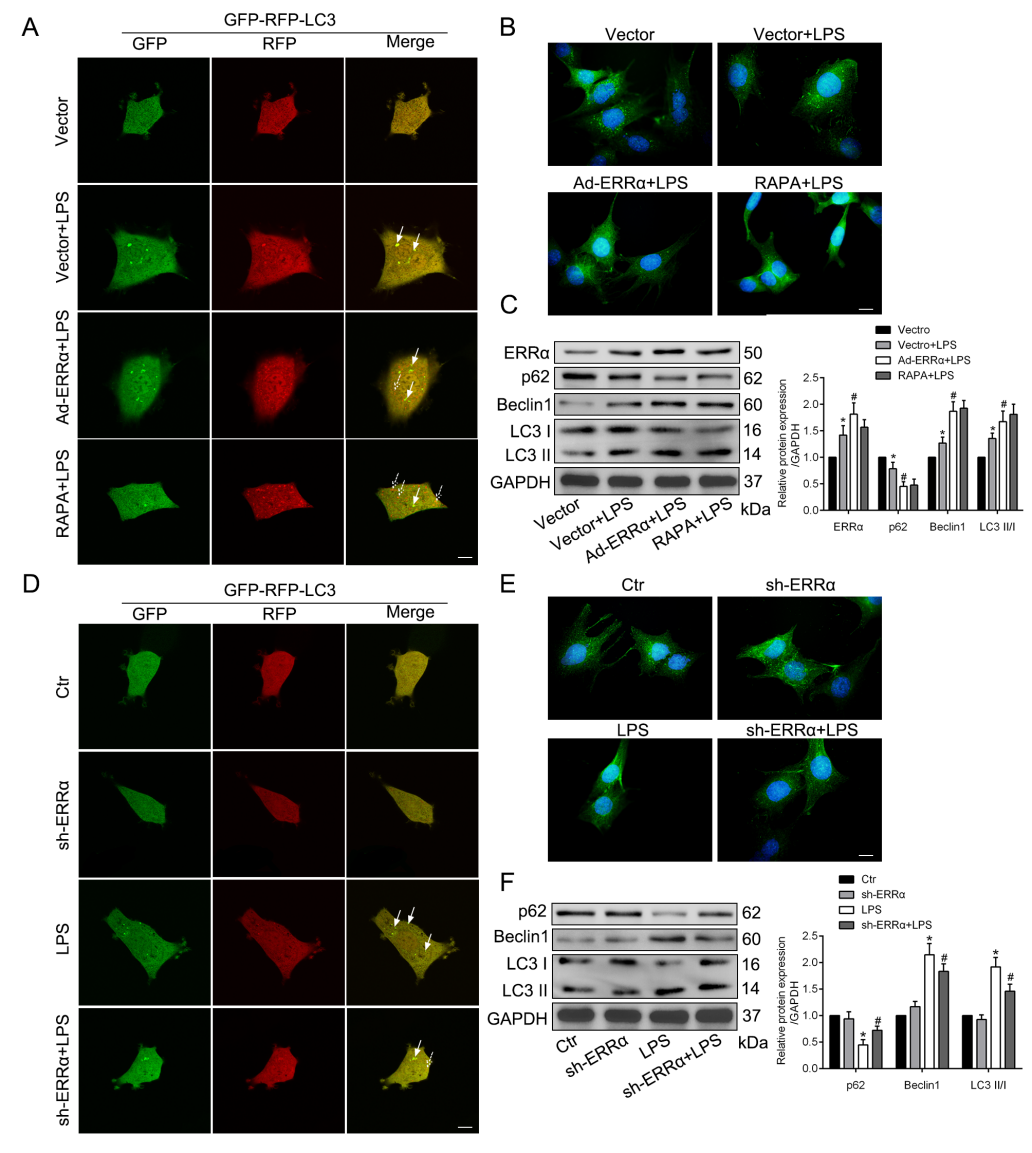
ERRα is essential for autophagy activation in PRMVECs in response to LPS. **A**, **D**. Representative confocal microscopy images of LC3 in different groups (**A**, the overexpressed adenovirus transfection groups and **D**, the knockdown lentivirus transfection groups) of PMVECs infected with RFP-GFP-LC3 adenovirus for 24 h. The yellow puncta (solid arrow) represent autophagosomes and the red puncta (dashed arrow) represent autolysosomes. Scale bar, 10 μm. **B**, **E**. Representative immunofluorescence showing the expression of p62 after LPS-treated from different groups (**B**, the overexpressed adenovirus transfection groups and **E**, the knockdown lentivirus transfection groups). Scale bar, 10 μm. **C**, **F**. Representative blots and quantitative analysis showing the relative protein levels of autophagy-associated proteins in different groups (**C**, the overexpressed adenovirus transfection groups and **F**, the knockdown lentivirus transfection groups). *P < 0.05 versus the Control or Vector group; #P < 0.05 versus the Vector + LPS or LPS group

**Fig. 4:**
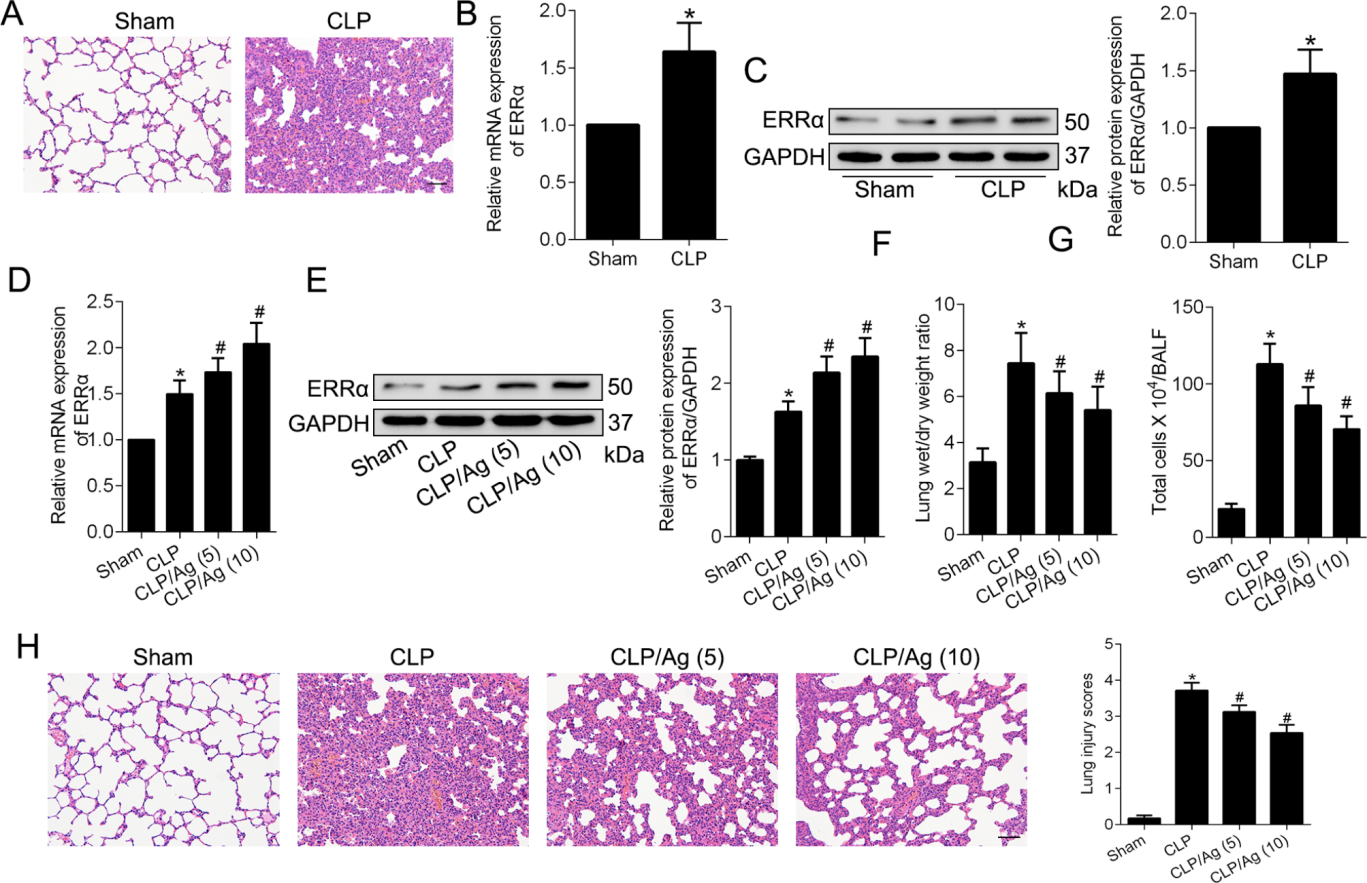
ERRα activation inhibits CLP-induced ALI in vivo. **A**. H&E staining showed morphological changes in lung tissues after CLP. Scale bar, 50 μm. **B**-**C**. RT-PCR (**B**), Western blot and quantitative analysis (**C**) of ERRα expression in Sham and CLP groups. **D**-**E**. RT-PCR (**D**), Western blot and quantitative analysis (**E**) showed that 16054-93-6 promoted the expression of ERRα in a dose-dependent manner in CLP-treated rats. **F**. Lung dry to wet weight ratio showed the degree of pulmonary edema. **G**. Total cell count in bronchoalveolar lavage fluid (BALF). **H**. Representative **H**&**E** staining micrographs showed the pathological damage of lung tissues in four groups as indicated. Scale bar, 50 μm. Values are expressed as mean ± SD. *P < 0.05 versus the Sham group; #P < 0.05 versus the CLP group

Kim, et al. found that ERRα is required for autophagy activation in macrophages in response to several autophagy inducers (Kim et al. 2018). As previous studies shown that autophagy responsively increased in LPS-induced ALI (Zeng et al. 2017; Liu et al. 2018), our study indicated that autophagy was enhanced in PMVECs after exposure to LPS. In order to confirm autophagy induction, we constructed a tandem of RFP-GFP-LC3 adenovirus. The yellow puncta represent autophagosomes and the red puncta represent autolysosomes. The successful transfection of the RFP-GFP-LC3 adenovirus was shown both fluorescent proteins (Fig. 3A, D). Compared with the control group, the red puncta were increased in PMVECs under LPS condition, which were more prominent in the ERRα overexpression group (Fig. 4A). Meanwhile, the protein levels of p62 in Ad-ERRα adenovirus-transfected cells were less than vector-transfected cells after LPS stimulation (Fig. 3B-C). We also observed that Beclin1 protein levels and the LC3B to LC3A ratio were significantly enhanced in ERRα overexpression group than that in control group after LPS treatment (Fig. 3C). Moreover, ERRα overexpression and RAPA had similar regulatory effects on LPS-induced autophagy. In contrast, ERRα deficiency inhibited the process of LPS-induced autophagy process (Fig. 3D-F).

### Promoting ERRα inhibits CLP-induced ALI in vivo

16054-93-6, a new identification agonist that promotes the transcriptional activity of ERRα (Peng et al. 2011), was utilized to explore the regulatory role of ERRα in CLP-induced ALI. Groups of rats were subjected to CLP, and treated with different doses of agonist via intraperitoneal injection. As shown in Fig. 4A-E, the mRNA and protein levels of ERRα were increased in rats after CLP and the agonist further improved the upregulation of ERRα expression. The results of W/D ratio, total cells count and H&E staining revealed that administration of agonist dose-dependently ameliorated leukocyte infiltration, pulmonary edema, destruction of alveolar septum, and thus alleviated ALI (Fig. 4F-H).

### Promoting ERRα expression reduces the CLP-induced degradation of junction protein and lung vascular hyperpermeability

**Fig. 5:**
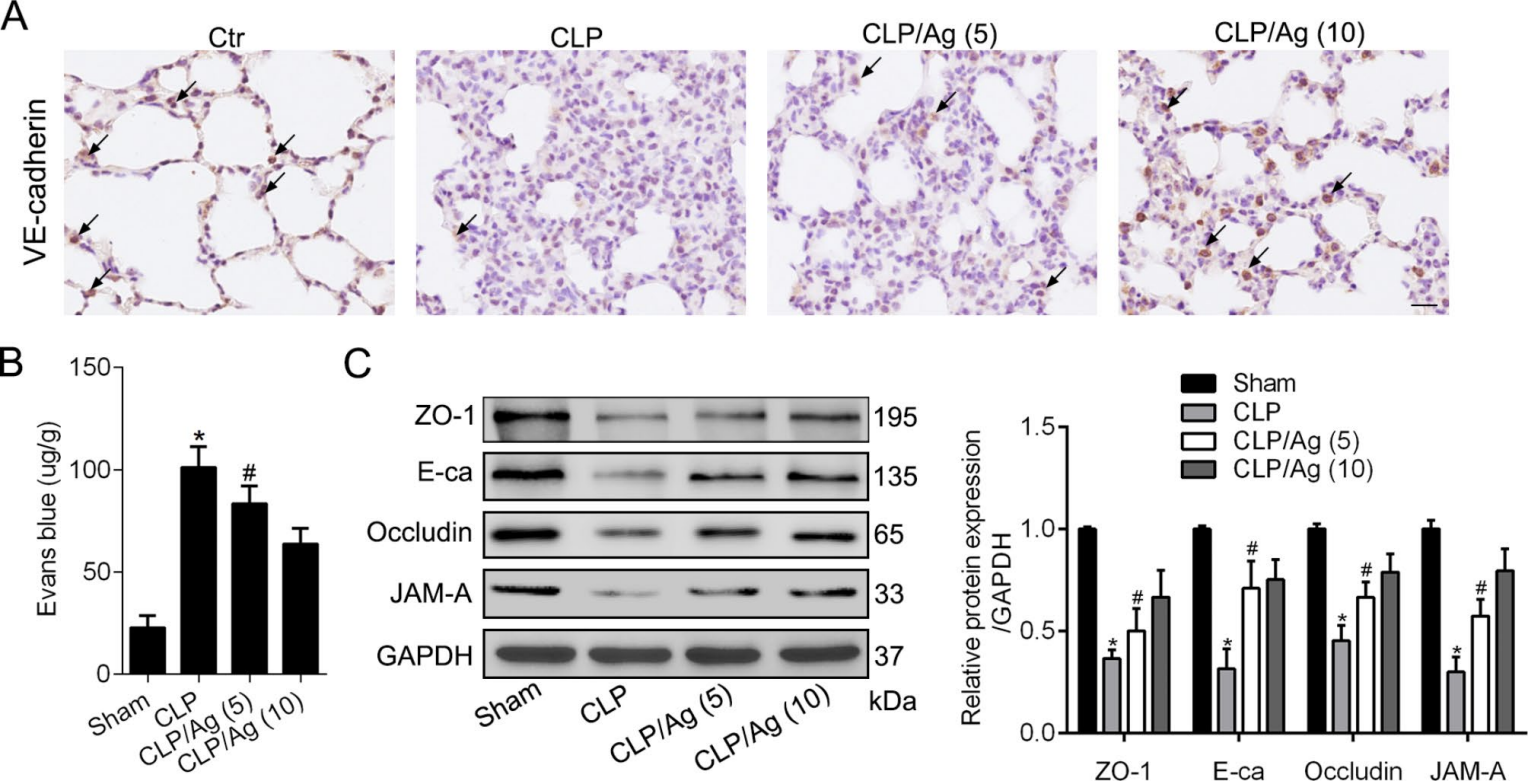
Promoting ERRα expression reduces the CLP-induced degradation of junction protein and lung vascular hyperpermeability. **A**. Representative micrograph of IHC showed lung VE-cadherin expression (solid arrow) in four groups as indicated. Scale bar, 25 μm. **B**. Pulmonary vascular permeability was compared by Evans blue dye extravasation in four groups. **C**. Western blot and quantitative analysis of ZO-1, Occludin, JAM-A, and VE-cadherin in the four groups. Values are expressed as mean ± SD. *P < 0.05 versus the Sham group; #P < 0.05 versus the CLP group

Impairment of adherens junctional integrity is a major feature of sepsis-induced ALI. Therefore, we also elucidated the effect of 16054-93-6 on lung adherens junction in sepsis-induced ALI. IHC shown that the major adherens junction protein, VE-cadherin, was markedly decreased in the lung tissues after CLP. ERRα agonist lessened the degradation of VE-cadherin in a dose-dependent manner (Fig. 5A). Collect BALF after injecting Evans blue dye in CLP-treated rats to assessed vascular permeability. Less Evans blue dye were found in BALF from CLP rats after agonist administration (Fig. 5B). Consistently, western blots shown that promoting ERRα expression ameliorated the degradation of tight junction proteins ZO-1, Occludin, JAM-A, and adherens junction protein VE-cadherin in the injured lung tissues of septic rats (Fig. 5C).

### ERRα has a dual protective effect in CLP-induced ALI

**Fig. 6:**
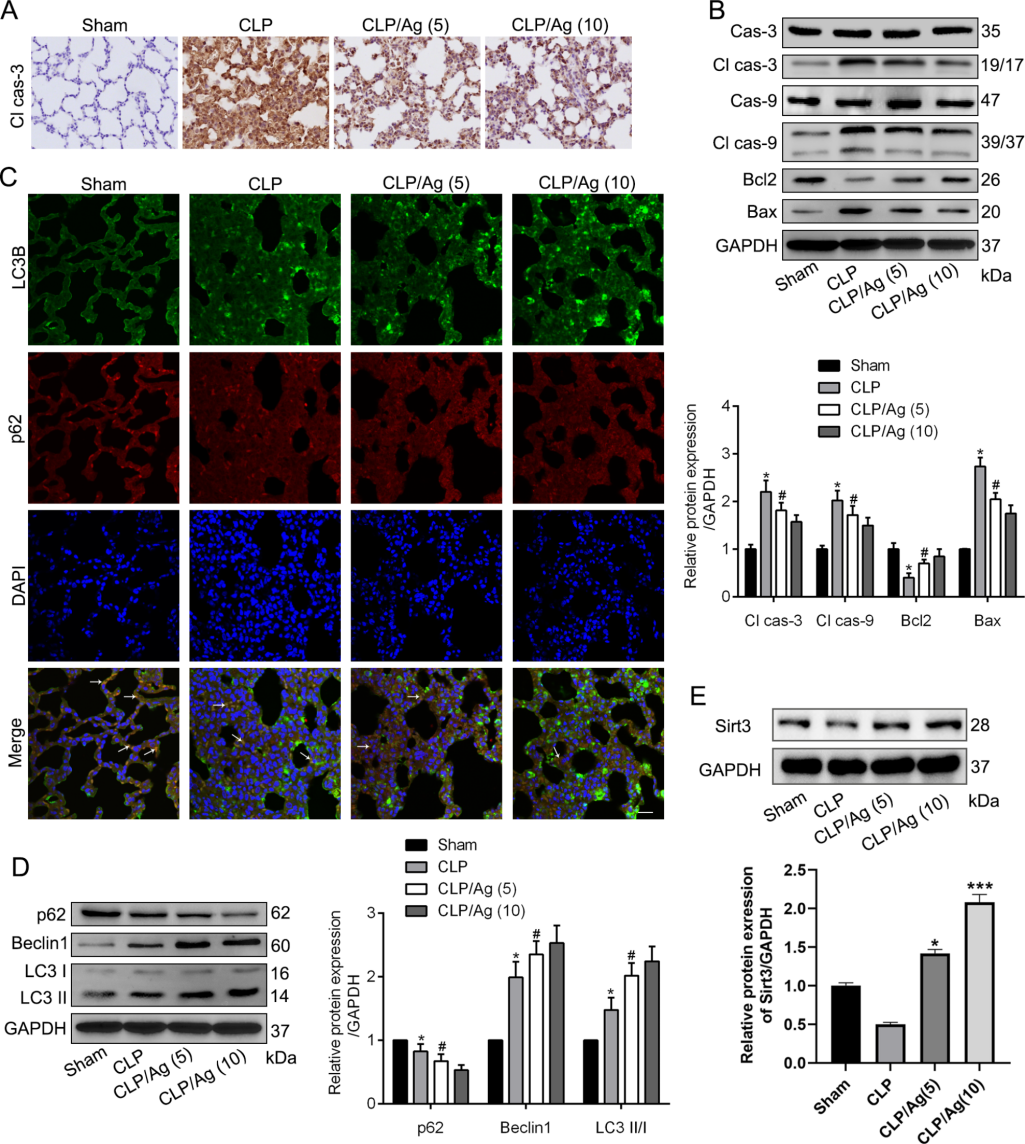
ERRα has a dual protective effect in CLP-induced ALI. Representative micrographs of IHC showed lung cleaved caspase 3 expression in four groups as indicated. Scale bar, 25 μm. **B**. Representative blots and quantitative analysis showing the relative protein levels of apoptosis-associated proteins in different groups. **C**. Immunofluorescence showing the changes of LC3B (green) and p62 (red) expression from agonist-treated rats after CLP, observed by confocal microscopic images. White arrows indicate colocalization expression. Scale bar, 50/3µm. **D**. Representative blots and quantitative analysis showing the relative protein levels of autophagy-associated proteins in different groups. **E**. Representative blots and quantitative analysis showing the expression levels of Sirt 3 in different groups. Values are expressed as mean ± SD. *P < 0.05 versus Sham group; #P < 0.05 versus the CLP group

In vivo, the ability of ERRα to suppress the apoptosis and improve autophagy was evaluated in CLP-treated rats. Agonist-treated rats exhibited dose-dependently decrease in the expression of cleaved caspase 3 (Fig. 6A) and apoptosis-related protein Bax (Fig. 6B), and significant increase in the levels of protein Bcl-2 (Fig. 6B) compared with the control rats after CLP. The immunofluorescence of p62 and LC3B in lung tissues shown that, under normal circumstances, p62 and LC3B proteins shared the same localization in cytoplasm (white arrow in Fig. 6C), and the levels of p62 were more prominent. However, After CLP, p62 epression decreased and LC3B expression increased (Fig. 6C). Furthermore, the protein expression of Beclin1 and the ratio of LC3B/A were increased in CLP-treated rats, which were further improved by ERRα agonist (Fig. 6D). Consistent with the results of the in vitro study, ERRα agonist significantly upregulated the expression of Sirt 3 in septic rats (Fig. 6E). These results confirmed that ERRα, overexpressed by 16054-93-6, suppressed apoptosis and induced autophagy in CLP-induced ALI.

## Discussion

In present study, the role and underlying mechanisms of ERRα in sepsis-induced ALI were investigated. The results showed that the overexpression of ERRα mitigated the LPS-induced degradation of adherens junction, enhancement expression of apoptosis-related proteins and promotion of autophagy induction. Conversely, deficiency of ERRα caused the opposite effect in PMVECs. ERRα agonist, 16054-93-6, upregulated the expression of junction protein through the suppression of apoptosis and the induction of autophagy, and thereby alleviated sepsis-induced ALI. The results of this study indicated that ERRα plays a vital role in the pathogenesis of sepsis-induced ALI and that ERRα is a potential therapeutic target for ALI.

The balance between oxidative and antioxidant systems is essential for maintaining intracellular homeostasis. When the balance between oxidation and antioxidant is disrupted under pathological conditions, the excessive accumulation of ROS will cause the activation of redox-sensitive transcription factors (such as NF-κB), which will promote intracellular inflammation and apoptosis (Lee and Choi 2003). Previous study reported that miR-34b-5p deficiency exerted protective effects via alleviating inflammatory response and epithelial cell apoptosis in LPS-induced ALI mice (Xie et al. 2018). In addition, Huang et al.(Huang et al. 2020) shown that ERRα upregulation promoted the expression of Bcl2 and inhibited caspase 3, which are the key regulators participating in cellular apoptosis, while inhibited ERRα by XCT790 can promote apoptosis of endometrial cancer cells. Wang et al.(Wang et al. 2017) found that PGC-1α upregulated the level of Bcl-2 to inhibit apoptosis and promote the survival of MSCs via PGC-1α/ERRα interaction. The mitochondrial apoptosis-induced channel (MAC) is an early marker of the onset of apoptosis, and MAC is regulated by Bcl-2 protein family: the pro-apoptotic molecule Bax form MAC, whereas the anti-apoptotic protein like Bcl-2 or Bcl-xL prevent MAC formation (Dejean et al. 2006). Once formed, MAC mediates the release of Cytochrome c to the cytoplasm. Cytochrome c binds with apoptotic protease activating factor-1 (Apaf-1) ATP, and then cleaves the pro-enzyme of caspase-9 to form apoptotic bodies triggering the apoptotic cascade (Li et al. 1997). In this study, we found that LPS induced the downregulation of Bcl2 and increase of cleaved-caspase 3 and cleaved-caspase 9 in vitro and in vivo, and the induction of apoptosis signaling was alleviated by ERRα overexpression, indicating that inhibition of apoptosis plays a pivotal role in the protect role of ERRα overexpression in ALI.

Autophagy, a process of recycling unnecessary components of cells under stress, plays a pivotal role in the development of many diseases. Dong et al. found that RAB26 maintained adherens junction stabilization in LPS-induced ALI via promoting the interaction between SRC and the autophagy marker LC3B (Dong et al. 2018). In addition, previous study found that mTOR deficiency significantly attenuated leukocyte infiltration, alveolar collapse and lung edema via augmenting autophagy, and displayed pulmonary protect effect in response to LPS treated (Hu et al. 2016). These studies have highlighted the evidence or the beneficial effect of autophagy to sepsis-induced ALI. Meanwhile, Kim et al. (Kim et al. 2018) demonstrated that ERRα plays an important role in the clearance of listerial and bacterial infection through modulating the host defense. They noted that ERRα interacts with Sirt1 to provoke the activation of autophagy through the deacetylation of autophagy-related genes, which enhances antibacterial effect and against mycobacterial infections. Moreover, ERRα served as a crucial molecule of intestinal homeostasis by activating autophagy and maintaining stability of host gut microbiota (Kim et al., 2021). Consequently, we speculated that ERRα may play an important function in the protection role of sepsis-induced ALI via the activation of autophagy. Herein we demonstrated that ERRα-mediated activation of autophagy reduced the hyperpermeability induced by LPS in vitro and in vivo. Overexpression of ERRα by Ad-ERRα adenovirus or agonist upregulated the expression of autophagy-related protein Becling1, LC3B and decreased the levels of SQSTM1/p62.

Maintaining homeostasis between apoptosis and autophagy is crucial for a cell to determine its fate in pathophysiological process. Autophagy and apoptosis are regulated by common signaling pathway in response to certain stimulation and show a certain degree of mutual inhibition. Beclin1 which interacted with Bcl2 and cleaved by activated caspase 3 (Murthy et al. 2014), is the key determinant of whether cells undergo apoptosis or autophagy. Under the sustains stimulated of apoptosis inducer, Beclin1 is cleaved to N- and C-terminal fragments by cleaved caspase 3, which is predominant mediator participated in apoptosis and autophagy. The Beclin1 N- and C-terminal fragments translocate to the mitochondria, invalidating the ability to induce autophagy of Beclin1 and provoking cell apoptosis (Lu et al. 2019). In addition, Lou et al. (Luo and Rubinsztein 2010) fund that Bax-induced apoptosis can reduce autophagy through elevating caspase-mediated cleavage of Beclin1 at D149. Subsequent study revealed that Vitamin D receptor deficiency increases apoptosis and degrades autophagy-relative protein ATG16L1 and Beclin1 and decrease the formation of autophagosome (Lu et al. 2019). Our report indicated that upregulating of ERRα protein decreased the level of cleaved caspase 3, cleaved caspase 9, Bax and promoting the expression of Beclin1, which were important to maintain the balance of apoptosis and autophagy. ERRα acts to increase the cell viability by promoting autophagy, reducing inflammation and cell apoptosis, and therefore maintaining the integrity of basement membrane of pulmonary endothelial cells.

Accordance with previous studies (Xia et al. 2020; Yuk et al. 2015), ERRα expression was upregulation in cells exposed to LPS or CLP-treated rats, the further expression of which plays a protective role in inflammatory induced by LPS. The early phase of inflammation requires more energy, which is mainly supplied by glycolysis and tricarboxylic acid cycle, while ERRα plays a predominant role in the positive regulation of mitochondrial energy metabolism. Therefore, it is understandable that ERRα responsiveness increases within 24 h after LPS or CLP treated. Our research also proved that 16054-93-6 has a protective effect in CLP-induced rats via improving the transcriptional functions of ERRα. The chemical structure of 16054-93-6 is similar to genistein, which not only has pleiotropic protective actions (Mukund et al. 2017), but also enhance the transcription activity of ERRα unselectively as a member of isoflavones (Suetsugi et al. 2003).

## Conclusion

Collectively, our results illustrate that ERRα protects against sepsis-induced ALI, at least in part by regulating the balance between autophagy and apoptosis to maintain the adherens junctional integrity. Genetic ablation of ERRα exacerbates apoptosis and inhibits the activation of autophagy in LPS-treated PMVECs. Overexpression or pharmacologic promotion of ERRα ameliorates damage in vitro or in vivo. Thus, the regulation mechanism of ERRα provides a new idea for the treatment of ALI.

## Data Availability

The datasets generated and/or analyzed during the current study are available from the corresponding author on reasonable request.

## Abbreviations

ALI: acute lung injury
ERRα: estrogen-related receptor alpha
LPS: lipopolysaccharides
PMVECs: pulmonary microvascular endothelial cells
CLP: cecal ligation and puncture
PGC-1α: peroxisome proliferator-activated receptor-c coactivator 1α
ROS: reactive oxygen species
TUNEL: TdT-mediated dUTP Nick End Labeling
IHC: immunohistochemical
BALF: Bronchoalveolar lavage fluid
Apaf-1: apoptotic protease activating factor-1
